# Recent Advances in Metal–Organic Frameworks (MOFs) and Their Composites for Non-Enzymatic Electrochemical Glucose Sensors

**DOI:** 10.3390/bioengineering10060733

**Published:** 2023-06-19

**Authors:** Panpan Li, Yi Peng, Jinpeng Cai, Yang Bai, Qing Li, Huan Pang

**Affiliations:** 1 Guangling College, Yangzhou University, Yangzhou 225009, China; 2 School of Chemistry and Chemical Engineering, Yangzhou University, Yangzhou 225009, China; 3 School of Pharmacy, Changzhou University, Changzhou 213164, China; 4 State Key Laboratory of Coordination Chemistry, Nanjing University, Nanjing 210008, China

**Keywords:** metal–organic frameworks, composites, non-enzymatic electrochemical glucose sensors

## Abstract

In recent years, with pressing needs such as diabetes management, the detection of glucose in various substrates has attracted unprecedented interest from researchers in academia and industry. As a relatively new glucose sensor, non-enzymatic target detection has the characteristics of high sensitivity, good stability and simple manufacturing process. However, it is urgent to explore novel materials with low cost, high stability and excellent performance to modify electrodes. Metal–organic frameworks (MOFs) and their composites have the advantages of large surface area, high porosity and high catalytic efficiency, which can be utilized as excellent materials for electrode modification of non-enzymatic electrochemical glucose sensors. However, MOFs and their composites still face various challenges and difficulties that limit their further commercialization. This review introduces the applications and the challenges of MOFs and their composites in non-enzymatic electrochemical glucose sensors. Finally, an outlook on the development of MOFs and their composites is also presented.

## 1. Introduction

Diabetes is a deadly disease that claims a large number of lives worldwide each year and causes several other conditions (e.g., kidney failure, stroke and blindness) [1,2]. Diabetes is a global epidemic at all income levels, especially in the world’s middle-income countries. According to the World Health Organization (WHO), the number of adults with diabetes quadrupled from 1980 to 2016, reaching 422 million [3]. As of 2019, there are 463 million people with diabetes worldwide, a number which is predicted to rise to about 578 million cases by 2030 and 700 million cases by 2045 [4,5]. There are generally two types of diabetes. Type 1 is caused by insufficient insulin production by the pancreatic islet cells, and type 2 is the result of the body not making enough use of the insulin produced by the pancreas. Although diabetes is a chronic disease, it can cause complications and severe symptoms, resulting in a large number of deaths [6,7]. In order to save lives and avoid diabetes-related diseases, it is essential to maintain blood sugar within the normal concentration range [8]. Fortunately, extensive research has shown that rigorous blood glucose monitoring can delay the development of diabetes-related complications and thus significantly reduce diabetes-related mortality [9,10]. Therefore, it is very urgent and necessary to use low-cost, simple and portable equipment to develop an accurate detection method for glucose concentration in fluids of the human body for patients to monitor and self-manage their blood glucose level [11,12].

Several glucose sensing devices and methods based on electrochemical, chemical and optical techniques have been produced in the last two decades. The electrochemical system has gained much attention because of high sensitivity, low detection limit, portability, low cost, convenient operation and good selectivity. Self-checking blood glucose electrochemistry was introduced in the 1980s. To date, it has achieved great success [13]. Electrochemical sensors, mainly on the basis of amperometric methods, are the most common class of glucose biosensors, including enzymatic and non-enzymatic sensors. Non-enzymatic amperometric glucose sensors are designed on the basis of direct electrochemical oxidation of glucose [14,15]. Despite their high selectivity and sensitivity, the enzyme-based glucose sensors also have certain drawbacks due to the presence of enzymes in their structure. For instance, the complex immobilization steps for enzymes make it difficult to guarantee the exact amount of enzyme, and enzymes are easily affected by temperature and PH value, making it difficult for enzyme sensors to be widely employed. Compared with enzymatic sensors, non-enzymatic electrochemical glucose sensors have attracted more and more attention because of their simple preparation, low environmental impact and superior stability [16].

Currently, precious metal catalysts such as platinum are the main material for assembling non-enzymatic electrochemical glucose sensors designed for direct detection of targets in blood samples. On the surface of platinum, glucose is oxidized to glucolactone by losing two electrons [17]. To enhance the detection performance of platinum-based glucose sensors, different methods have been adopted [18]. However, a number of challenges still stand in the way of their use in practical blood analysis sensors (e.g., poor selectivity and low stability) [19]. In addition, the overall kinetics of glucose electrooxidation remain too sluggish to provide a significant amperage response [20]. The electrodes commonly used in electrochemical glucose sensors include the glassy carbon electrode (GCE) [21,22], carbon paste electrode (CPE) [23,24], indium tin oxide electrode [25,26], etc. These electrodes are usually less sensitive to glucose and need to be modified to improve their performance. Therefore, scientists are exploring novel materials with low cost, high stability and excellent performance to carry out chemical modification of non-enzyme electrochemical glucose sensor electrodes.

Metal–organic frameworks (MOFs), comprising metal nodes and organic ligands, are surprisingly porous materials with strong functionality, high porosity, large specific surface area, adjustable pore size, biomimetic catalysis and biocompatibility [27,28,29,30]. MOFs are promising materials for use in electrochemical glucose sensors for several reasons [31]: On the one hand, due to the unique structural advantages (e.g., large surface area, adjustable porosity and cavity structure), periodic network structure and unsaturated metal coordination, MOFs have superior catalytic capacity and efficient concentration and mass transfer capacity of analytes, allowing effective amplification of signal responses and enhanced detection sensitivity. Therefore, MOFs can be used in sensing applications as effective electrocatalytic coating materials to modify the electrodes and improve their sensitivity to glucose. On the other hand, the specific size and shape of available channels and cavities give the MOF-based materials good selectivity for specific analytes via the size exclusion effect [32,33]. Thanks to these advantages, a series of pristine MOFs with excellent electrical activity were directly used as electrocatalysts to detect glucose [34,35].

**Scheme 1:**
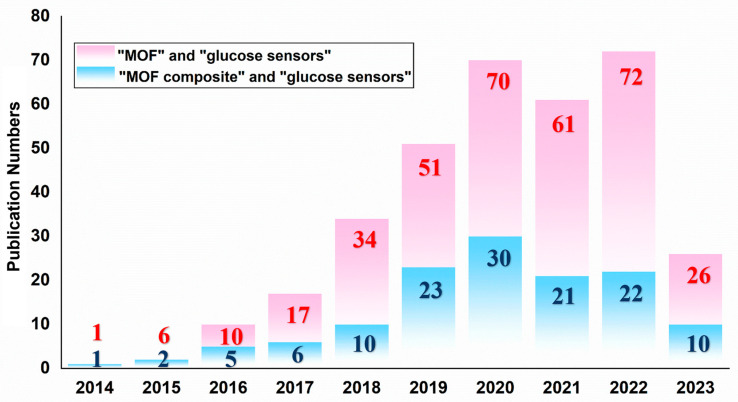
Number of MOF-based and MOF composite-based glucose sensor-related articles published in the past 10 years (up to 1 June 2023), obtained from Web of Science.

**Scheme 2:**
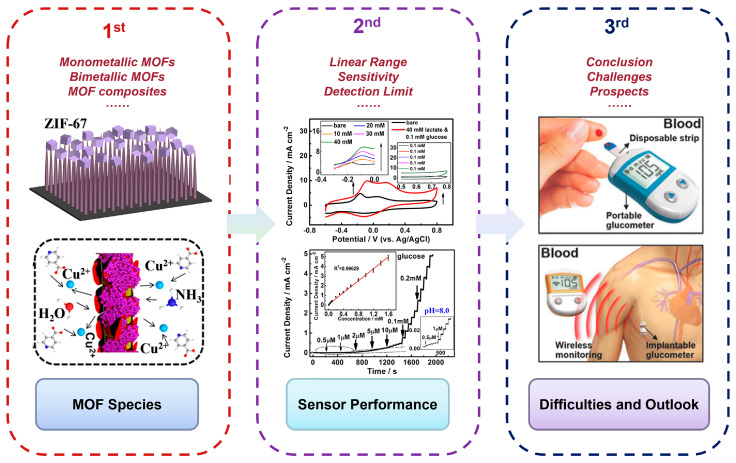
Graphical abstract of the introduction of MOFs and their composites in this review.

Despite these attractive properties of pristine MOFs, the low conductivity and poor stability hinder their applications [36,37]. MOFs can be facilely coupled with functional materials to prepare MOF composites with more efficient electrochemical reactivity rates and higher electrical conductivity [38,39,40]. In addition, MOF derivatives (e.g., metal oxides/carbon composites) have also attracted much attention for their rich internal voids and good electrical conductivity [41,42,43,44]. However, during the derivation of MOFs, the structure of MOFs can collapse unexpectedly, weakening their advantage of large surface area. Therefore, in recent years, more and more studies on MOF-based non-enzyme electrochemical glucose sensors have been focused on MOF composites (Scheme 1). However, there are no specific reviews of the applications of MOFs and their composites in non-enzymatic electrochemical glucose sensors. Herein, as shown in Scheme 2, the advanced research progress of pristine MOFs and their composites in non-enzymatic electrochemical glucose sensors as modified materials for electrodes is reviewed. Additionally, the existing difficulties and future development trends of advanced MOF-based non-enzymatic electrochemical glucose sensors are discussed.

## 2. Pristine MOFs as Modified Materials for Electrodes

The unique framework and porous structure of pristine MOFs creates a rich exposure space for functional elements that can act as catalytic centers for glucose sensing to modify electrodes. Exposure to massive active sites in pristine MOFs results in superior electrocatalytic performance, such as exceptional sensitivity and low detection limit [45]. More importantly, due to the compatibility of pristine MOFs with the pore size of glucose molecules, the molecular sieve effect of pristine MOFs in the sensing process overcomes the problems of low selectivity in non-enzymatic catalysis [46]. There are many methods for adjusting the size, morphology and sensitivity of the pristine MOFs, including using functional organic ligands with amido-functional groups, carboxy groups and sulfonic acid groups [47], which can not only regulate the surface morphology but also provide electrical conductivity in pristine MOFs and enhance the electrochemical adsorption of glucose molecules [48]. In addition, the synergistic effect of two or more components can also improve the electrochemical performance. That is, the electrocatalytic performance of monometallic MOFs can be improved by synthesizing MOFs with uniform distribution of two or more metal elements and well-controlled morphology [49,50,51].

### 2.1. Monometallic MOFs

#### 2.1.1. Co-Based MOFs

**Figure 1:**
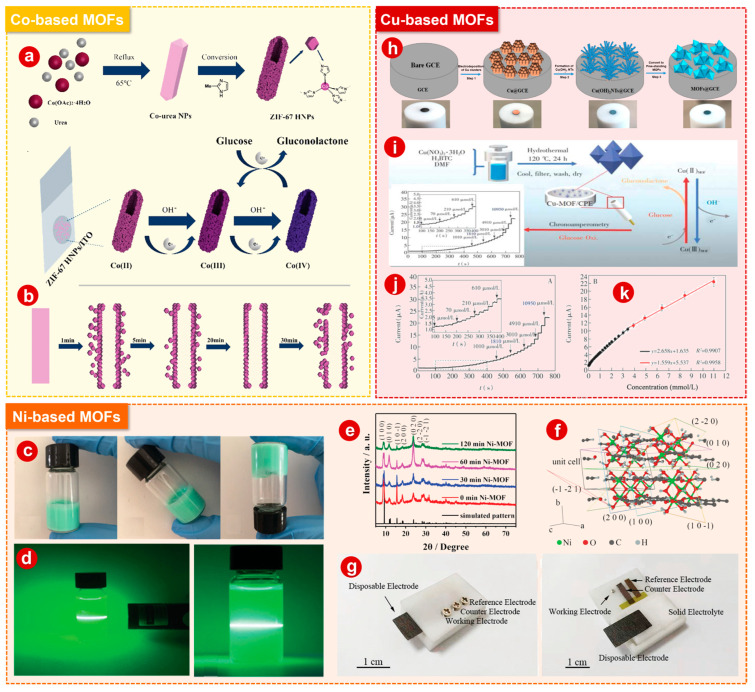
(**a**) Schematic diagram of preparation of ZIF-67 HNPs and mechanism of glucose electrooxidation under ZIF-67 HNPs/ITO. (**b**) Schematic diagram of ZIF-67 HNP formation process. (**c**) Images of self-supporting hydrogels. (**d**) Tyndall effects. (**e**) XRD patterns of Ni-MOFs under ultrasonic treatment at 0, 30, 60 and 120 min. (**f**) Corresponding crystal planes in Ni-MOFs. (**g**) Photos of all-solid-state non-enzymatic sweat glucose biosensor device. (**h**) Schematic diagram of free-standing Cu-based MOF thin films prepared on GCE. (**i**) Schematic diagram of synthesis of Cu-MOF catalyst and electrochemical glucose sensing principle. (**j**) Amperometric response of Cu-MOF/CPE to the successive addition of glucose. (**k**) The corresponding calibration curves. Panels (**a**,**b**): Reprinted with permission [56]. Copyright 2021, Royal Society of Chemistry. Panels (**c**,**d**): Reprinted with permission [62]. Copyright 2017, Royal Society of Chemistry. Panels (**e**–**g**): Reprinted with permission [64]. Copyright 2020, Royal Society of Chemistry. Panels (**h**): Reprinted with permission [68]. Copyright 2018, Elsevier. Panels (**i**–**k**): Reprinted with permission [69]. Copyright 2020, Elsevier.

In 1995, Professor Omar M. Yaghi’s group at UC Berkeley synthesized a coordination compound called an MOF with a two-dimensional (2D) structure using benzene-1,3,5-tricarboxylic acid and Co(NO_3_)_2_, which was published in the journal *Nature* [52]. Since then, the concept of metal–organic frameworks has been formally proposed [53,54]. Co-based MOFs have the advantages of adjustable active site rule, porous structure, etc. [55]. ZIF-67 hollow nanosheets (ZIF-67 HNPs) were prepared by Chen et al. [56] by a simple and effective diffusion-controlled method for non-enzymatic glucose sensors (Figure 1a). A schematic diagram of the ZIF-67 HNP formation process is shown in Figure 1b. This nanostructured Co-based MOF sensor delivers excellent electrocatalytic activity against glucose oxidation in alkaline solutions with a sensitivity as high as 445.7 μA mM^−1^ cm^−2^, a low detection limit of 0.96 μm (S/N = 3), as well as an upper detection limit of 42.1 mM.

#### 2.1.2. Ni-Based MOFs

Since Ni^2+^/Ni^3+^ redox couples are easily formed, nickel-based non-enzymatic glucose sensors also benefit from this property, and Ni-based MOFs are able to take full advantage of their active site and provide adequate glucose sensing catalysts [57,58]. In addition, due to the large specific area and large pore size of Ni-based MOFs, they are a suitable candidate for electron transport and target material diffusion. Ni-based MOFs with abundant pores and catalytic active sites can make a rapid response to target substrates and thus exhibit excellent electrochemical performance [59,60]. In general, benefiting from the above advantages, Ni-based MOFs exhibit better electrochemical properties than other MOFs, for example, Gilang Gumilar and coworkers [61] prepared some MOFs with different coordination metal ions, among which the Ni-based MOF showed the best electrochemical performance, and investigated its potential in electrochemical glucose sensing. Our group [62] developed a self-supporting gel (Figure 1c) that imparts ultrathin Ni-MOF nanobelts with powerful functional molecular encapsulation capabilities. In addition, the Tyndall effect (Figure 1d) directly confirms the product is a gelatin system. The ultrathin Ni-MOF nanobelt/GCE has superior performance, a wide linear range from 1 to 500 μM, low detection limit of 0.25 μm (S/N = 3), as well as a response sensitivity of 1.542 μA^−1^ cm^−2^. In addition, the sensor has a high accuracy in measuring real samples with a relative standard deviation of 7.41% in the detection of glucose in human serum. Lopa et al. [63] were the first to use CPO-27-Ni^II^ for non-enzymatic detection of glucose. CPO-27-Ni^II^ has excellent stability in water, and its pore size is around 1.1 nm, allowing glucose molecules to enter or adsorb into its pores. Wide linear range, high sensitivity of 40.95 μA mM^−1^ and low detection limit of 1.46 μM are all advantages of CPO-27-Ni^II^-modified GCEs. Xuan et al. [64] used ultrasound-induced longitudinal expansion of Ni-MOFs to increase active Ni cation sites to address the lack of active Ni cation sites in the stacking MOF layers, which enhanced the current response to glucose detection. The X-ray diffraction (XRD) patterns of Ni-MOFs under ultrasonic treatment at 0, 30, 60 and 120 min are shown in Figure 1e, and it can be observed that, as the sonication time increases, due to the expansion of Ni-MOFs along the b-axis after sonication, the intensity of some of the planes (e.g., (1 0 0), (2 0 0), (1 0 1)) decreases and the intensity of some of the planes (e.g., (0 1 0), (0 2 0)) increases [65]. The intensities of the (0 1 0) and (0 2 0) planes increased from 0 to 60 min, but further decreased at 120 min due to the complete collision and disorder of these peel planes caused by extended ultrasound exposure. Ni-MOF, after 60 min of ultrasonic treatment, showed the best performance for non-enzymatic electrochemical glucose sensors with a sensitivity up to 3297.10 μA mM^−1^ cm^−2^, a detection limit as low as 8.97 μM (S/N = 3), a wide linear response ranges from of 10 to 400 μM and a sensitivity of 3.03 μA mM^−1^ cm^−2^. In addition, as shown in Figure 1g, an all-solid-state glucose biosensor assembled with a solid electrolyte and Ni-MOF working electrode was also explored for non-enzymatic sweat glucose detection.

#### 2.1.3. Cu-Based MOFs

Due to the wide range of oxidation states of Cu-based materials (e.g., Cu^0^, Cu^I^, Cu^II^ and Cu^III^), allowing for reactions via single- and double-electron pathways, a wide variety of reactions can be promoted [66]. Thanks to this, Cu-based materials have been applied in the field of electrochemistry, and representative Cu-based MOFs such as HKUST-1 have been born [67]. For the first time, Saeed Shahrokhian and colleagues [68] obtained surface-modified GCEs by directly growing Cu-based MOF films, developing a novel non-enzymatic glucose sensing platform that consists of two fast and highly controlled conversion steps (Figure 1h). Direct growth of Cu-based MOFs is achieved by forming vertically aligned copper clusters and copper hydroxide nanotube arrays, which can be used as mediators and locational fixation factors to rapidly form self-supporting MOFs on the surface of GCEs. Furthermore, the prepared MOF thin film electrode has the characteristics of uniform structure, good stability, good diffusion of analytes and electrolytes, etc. and can straightforwardly serve as electrode materials for non-enzymatic electrocatalytic oxidation of glucose. Lan et al. [69] developed an electrochemical voltammetry sensor for glucose by encapsulating Cu-MOFs in carbon paste to obtain Cu-MOFs modified with CPEs (Figure 1i). As shown in Figure 1j,k, the steady value of the current was reached within 0.5 s with good catalytic performance for glucose. Over the range of 5 to 3910 μM, the catalytic current showed a positive linear correlation with glucose concentration.

### 2.2. Bimetallic MOFs

**Figure 2:**
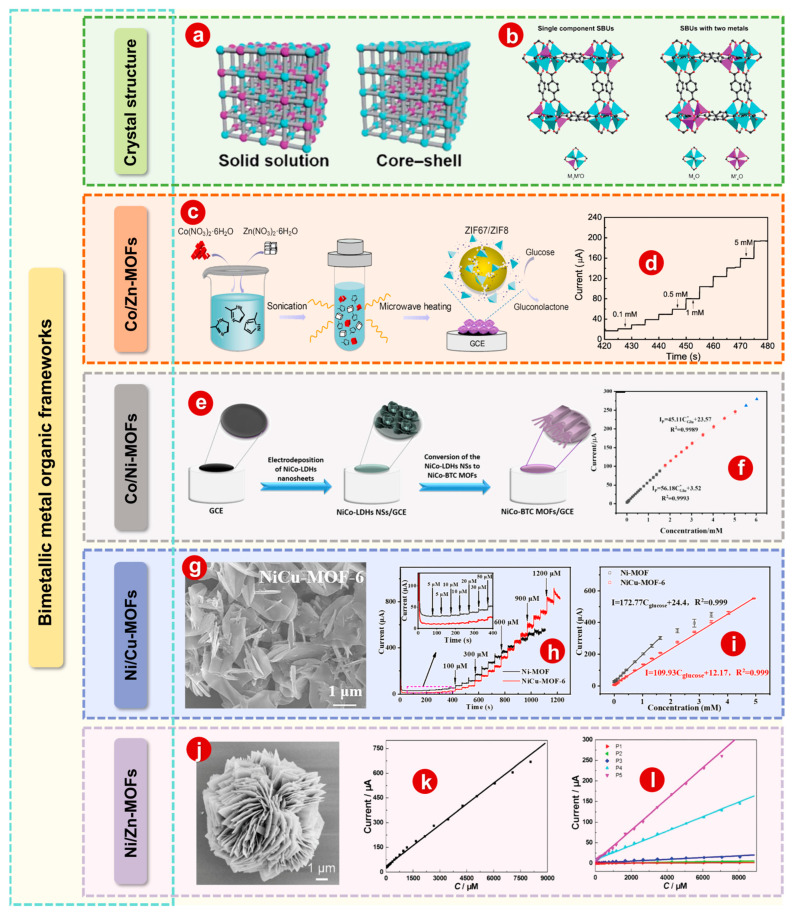
(**a**) The “solid solution” or “core–shell” structure of bimetallic MOFs. (**b**) Two spatial arrangements of metals in solid solution bimetallic MOFs and their SBUs. (**c**) Schematic diagram of one-step microwave-assisted synthesis of bimetallic Co/Zn MOFs for enzyme-free glucose detection. (**d**) Current–time curve of continuous glucose injection into electrolyte. (**e**) Schematic diagram of the preparation of NiCo-BTC MOFs/GCE. (**f**) Calibration curve of current response and glucose concentration with two linear ranges within 0.001–1.78 and 1.78–5.03 mM. (**g**) SEM image of NiCu-MOF-6. (**h**) Amperometric responses of Ni-MOF/GCE and NiCu-MOF-6/GCE upon successive addition of glucose. (Inset: magnified amperometric response to glucose at lower concentrations.) (**i**) The corresponding calibration curves of Ni-MOF/GCE and NiCu-MOF-6/GCE. (**j**) SEM image of Ni-MOF. (**k**) The corresponding calibration curve of Ni-MOF. (**l**) The corresponding calibration curve of Ni/Zn-MOFs. Panels (**a**,**b**): Reprinted with permission [51]. Copyright 2020, Royal Society of Chemistry. Panels (**c**,**d**): Reprinted with permission [79]. Copyright 2022, Elsevier. Panels (**e**,**f**): Reprinted with permission [80]. Copyright 2020, American Chemical Society. Panels (**g**–**i**): Reprinted with permission [83]. Copyright 2021, Elsevier. Panels (**j**–**l**): Reprinted with permission [84]. Copyright 2018, Royal Society of Chemistry.

Due to the synergistic interaction between the two metal atoms, the electronic structure of bimetallic MOFs is adjusted to enhance their catalytic activity [70,71]. The adjustable and even controllable ratio between the two metals makes it possible to modulate the physicochemical properties of bimetallic MOFs [72,73]. According to the distribution of metal ions, bimetallic MOFs are categorized into “solid solution” and “core–shell” structures (Figure 2a) [74]. For bimetallic MOFs with a “solid solution” structure, the metal is dispersed and uniformly distributed throughout the crystal. They can be classified into two categories according to the spatial arrangement of the metals (Figure 2b): (1) Two different metal ions in the same secondary building units (SBUs) and mixed metal SBUs run through the whole MOF structure; (2) the same metal makes up each SBU but two different SBUs are mixed well in the MOF structure. In addition, MOF shells are chemically different from MOF cores. These bimetallic MOFs are called “core–shell” bimetallic MOFs, and MOF shells and cores are integrated into a single structure [51]. Compared to their single-metal counterparts, bimetallic MOFs with adjustable composition and structure usually show enhanced performance in electrochemical sensors [75,76,77].

#### 2.2.1. Co/Zn-MOFs

To prepare Co/Zn-BTC MOFs directly on the surface of GCEs, Kachouei et al. [78] developed a fast and simple in situ two-step method by electrodeposition of Co/Zn-layered double hydroxide (LDH) as a sacrificial intermediate. The structural characterization of materials and the phase of each step are studied by many means. Compared to traditional synthesis methods of MOFs, the chemical reactions of this in situ two-step method are fast, requiring only about eight minutes to manufacture the entire electrode by applying negative potential to the work GCE without using any dangerous organic solvents or adhesives in preparation, making it environmentally friendly. This sensor has selectivity of peer structure and coexisting interference, high long-term persistence, fast response of 1.3 s, good resistance to toxicity, good repeatability and superior reproducibility of glucose electrooxidation. Furthermore, Li et al. [79] designed a convenient microwave-assisted method to prepare bimetallic Co/Zn MOFs (Figure 2c). The current–time curve with the successive injection of glucose is shown in Figure 2d. The synergistic effect between Co and Zn is critical for optimizing electrical analysis capabilities, which engenders excellent glucose sensing properties.

#### 2.2.2. Co/Ni-MOFs

By electrodeposition of CoNi-LDHs, bimetallic Co/Ni-MOFs with thin sheet structure were synthesized on the surface of GCEs by Milad Ezzati and colleagues (Figure 2e) [80]. The prepared electrodes have two linear dynamic ranges (Figure 2f) and showed good selectivity for interferents, high anti-toxicity for chloride ions and high repeatability and reproducibility of glucose electrooxidation, and the detection limit is 0.187 μM (S/N = 3). Our group [81] synthesized 2D bimetallic Ni/Co-MOF nanoplates by solvothermal synthesis modulated by pyridine, followed by surface modification using 4-halopyridine to overcome the drawbacks of pristine MOFs. NiCoBP-Br has the best sensing activity for the glucose oxidation reaction in the class of halogenated NiCo-MOF nanoplates, with a superior sensitivity of 1755.51 μA mM^−1^ cm^−2^, wide linear response range of 0.5 to 6065.5 μM and a quick response time of 2 s or less. In addition, there is almost no loss of response current after 6000 s of cycling. The study of NiCoBP-Br cycling for 12 h showed that the source of high electrocatalytic activity is the hydroxides formed during the reaction and the hydroxides and inherited hydroxides.

#### 2.2.3. Ni/Cu-MOFs

Xue et al. [82] prepared a new Ni/Cu-MOF with 2D nanosheet structure for oxidation of glucose with the following advantages: A wide linear range of 5 to 2500 μM, a low detection limit of 1.67 μM (S/N = 3), as well as a high sensitivity of 1703.33 μA mM^−1^ cm^−2^. Pan and colleagues [83] developed a series of 2D/3D layered bimetallic Ni/Cu MOFs by a one-step hydrothermal method. The optimal NiCu-MOF-6 (with mass ratio of Ni(NO_3_)_2_·6H_2_O/Cu(NO_3_)_2_·3H_2_O of 6) shows high electrocatalytic activity in glucose oxidation. As can be seen from Figure 2h,i, NiCu-MOF-6/GCE, as an electrode modifier for GCEs, has a significantly larger linear range than its single-metal analog due to its mass/electron transfer interconnecting channels and cofunctionalized electroactive sites of Ni/Cu.

#### 2.2.4. Ni/Zn-MOFs

Our group [84] explored the synthesis of Ni^2+^ and PTA to prepare ultrathin 2D nanosheet Ni-MOF layered flowers, and an SEM image is shown in Figure 2j. For as-prepared Ni-MOFs, a plot of the electrocatalytic current of glucose is shown in Figure 2k, and the results show that the catalyst has excellent electrocatalytic activity and stability in the concentration range of 0.5 to 8.065 µM. In addition, the improvement of electrocatalytic activity of Ni-MOF stratified flowers was also studied by controlling the amount of Zn. For as-prepared Ni/Zn-MOFs, a plot of the electrocatalytic current of glucose versus its concentrations in the 0.5 to 8.065 mM range is shown in Figure 2l, and the results show that the electrocatalytic activity of Ni/Zn-MOFs increases with the increase in Ni content.

## 3. MOF Composites as Modified Materials for Electrodes

In order to enhance the pivotal physicochemical performances of pristine MOFs, such as electrical conductivity and electrocatalytic property, pristine MOFs can be compounded with functional materials, including carbonaceous substrates, metal nanoparticles, conductive polymers, etc. [85,86,87,88,89]. These MOF composites have physicochemical properties that are not present in the pristine MOFs [90], as well as their own excellent electrochemical activity and the high stability conferred by other functional materials. In addition, novel advanced MOF structures can be realized by compounding carbon-based materials, metal nanoparticles, etc., thus obtaining improved porosity, regulated surface area and modified electrocatalytic properties [91]. These modifications of the MOF composites can better modify the electrode surface than the pristine MOFs [92,93].

### 3.1. MOF/Carbon-Based Composites

Pristine MOFs can be assembled with carbon-based conductive materials (e.g., graphene, reduced graphene (GO), carbon nanotubes (CNTs), carbon nanofibers (CFs), etc.) to prepare MOF/carbon-based composites. In this regard, carbon-based composites, as a unique conductive additive material, can improve the conductivity and mechanical strength of pristine MOFs [94,95]. In addition, MOFs and other carbon-based conductive materials have been applied to prepare novel composites with excellent electrochemical properties. Due to the above advantages, MOF/carbon-based materials have been widely used in electrode modification materials.

#### 3.1.1. MOF/Graphene

**Figure 3:**
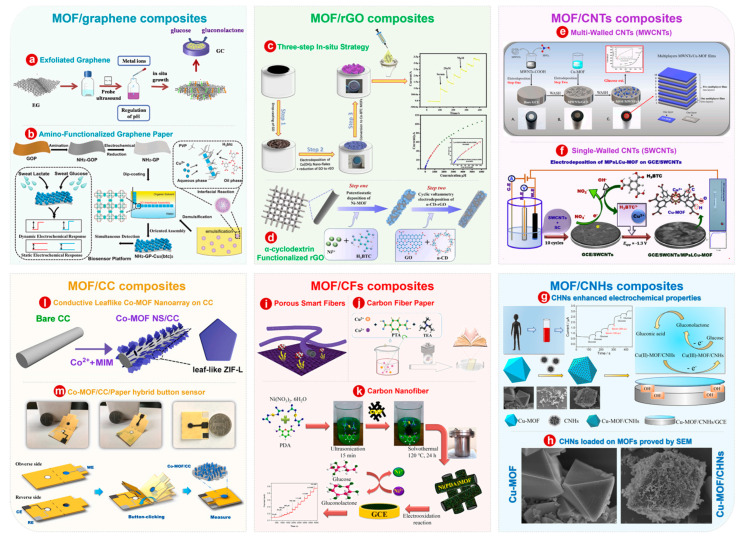
(**a**) Schematic diagram of the fabrication processes of MOF/exfoliated graphene. (**b**) Schematic diagram of the synthetic paths of the NH_2_-GP-Cu-MOF electrode. (**c**) Schematic diagram of the fabrication processes of Co-MOFs/rGO/GCE and electrochemical performance diagram. (**d**) Schematic diagram of the synthetic paths of α-CD-rGO/Ni-MOF/TM. (**e**) Schematic diagram of the Cu-MOF and multilayer films of Cu-MOF/MWCNTs/GCE. (**f**) Schematic diagram of the fabrication processes of MPsL Cu-MOF on SWCNTs/GCE by electrodeposition. (**g**) Schematic diagram of the synthetic paths of Cu-MOF/CNH-modified GCE and the application for glucose detection. (**h**) SEM images of Cu-MOF and Cu-MOF/CNHs. (**i**) Schematic diagram of functional smart fibers and corresponding functional textile. (**j**) Schematic diagram of the fabrication process of flaky book-like CuCo-MOF fixed on CF paper at 150 °C for 6 h. (**k**) Schematic illustration of formation process of Ni-MOF@CNF material and its application in glucose detection. (**l**) Schematic diagram for the synthetic paths of Co-MOF phase with well-aligned 3D nanosheet array architecture on CC. m) Photos of the button sensor (**Top**) and 3D diagram of the measurement process (**Bottom**). Panel (**a**): Reprinted with permission [99]. Copyright 2020, Elsevier. Panel (**b**): Reprinted with permission [33]. Copyright 2018, Royal Society of Chemistry. Panel (**c**): Reprinted with permission [103]. Copyright 2019, Elsevier. Panel (**d**): Reprinted with permission [104]. Copyright 2022, Elsevier. Panel (**e**): Reprinted with permission [111]. Copyright 2019, Elsevier. Panel (**f**): Reprinted with permission [112]. Copyright 2020, Elsevier. Panel (**e**): Reprinted with permission [111]. Copyright 2019, Elsevier. Panels (**g**,**h**): Reprinted with permission [117]. Copyright 2020, Elsevier. Panel (**i**): Reprinted with permission [120]. Copyright 2020, Royal Society of Chemistry. Panel (**j**): Reprinted with permission [121]. Copyright 2021, Royal Society of Chemistry. Panel (**k**): Reprinted with permission [122]. Copyright 2022, Elsevier. Panel (**l**): Reprinted with permission [131]. Copyright 2018, American Chemical Society. Panel (**m**): Reprinted with permission [132]. Copyright 2020, Elsevier.

Graphene is a novel material with sp² hybrid connected carbon atoms tightly packed into a single 2D honeycomb lattice structure [96]. Owing to its unique 2D structure and excellent physicochemical properties, graphene has rapidly aroused great interest in many disciplines [97,98]. Graphene is an excellent material, and the electrochemical performance of MOFs is enhanced though combining it with MOFs. Liu et al. [99] used an economical strategy to synthesize 2D MOFs with different metal nodes. Electrochemical exfoliated graphene was used, whose edge planes and defects facilitate electrochemical applications [100], to enhance the mechanical stability and electrical conductivity of 2D MOFs (Figure 3a). Due to the participation of exfoliated graphene, the modified MOF/exfoliated graphene electrode has high glucose sensing electrocatalytic activity when applied as the electrode material of a non-enzymatic glucose sensor. Among all the different metal nodes, Co-MOF/exfoliated graphene has the lowest oxidation potential for glucose, and the detection performance is the best at the low oxidation potential of 0.2 V. Modifying functional groups on graphene is also a way to improve its performance, for example, the hydrophilicity and polarity of the graphene surface can be improved by introducing amino groups on the graphene surface. In addition, the amino group on the surface of amino graphene has polycondensation reactivity and can be polymerized in situ with organic acid salts to prepare functional amino graphene composite. Wang et al. [33] prepared a MOF electrode on the basis of flexible amino-functionalized graphene paper (NH_2_-GP-Cu-MOF), which was modified by simple interfacial synthesis and an effective dip-coating method, as shown in Figure 3b. Wang et al. [33] also found that 2D oriented assembly of Cu-MOF nanocubes at the oil–water interface could be transferred to an amino-functionalized graphene paper, resulting in a dense, uniform monolayer of Cu-MOF nanocubes loaded onto the paper electrode.

#### 3.1.2. MOF/Reduced Graphene Oxide

As a form of graphene, reduced graphene oxide (rGO) has properties similar to graphene (good electrical conductivity, etc.), but contains more defects and is of lower quality than graphene produced directly from graphite [101]. rGO can be synthesized by a variety of reduction methods from graphene oxide (GO) [102]. In addition, the MOFs are compounded with rGO, rather than GO, because the oxidation process destroys the highly conjugated structure of graphene, resulting in the low conductivity of GO, which is not conducive to the transport of electrons. Therefore, GO is often used as a precursor to synthesize MOF composites and reduce GO to rGO.

Saeed Shahrokhian et al. [103] used a fast and easy in situ three-step method to develop a non-enzymatic sensor to measure glucose by controllable growth of Co-MOF thin films on the surface of GCEs (Figure 3c). The as-prepared electrode has a wide linear dynamic range, good repeatability and reproducibility, high selectivity for interfering substances and good resistance to chlorine ion poisoning, and the detection limit is as low as 0.33 μM (S/N = 3). rGO can improve the performance of MOFs. Whether the MOF/rGO composites as a whole have a performance-enhancing effect remains to be proven. As shown in Figure 3d, Xu et al. [104] developed a rapid and facile two-step electrodeposition method for α-cyclodextrin-functionalized rGO/Ni-MOFs on titanium mesh (α-CD-rGO/Ni-MOF/TM) composite membranes to build a non-enzymatic glucose sensing platform. Compared with pristine TM, α-CD-rGO/Ni-MOF/TM nanocomposites showed better glucose electrocatalytic activity. It is proved that MOF/rGO can also improve the electrochemical performance of certain materials. Of course, multiple functional materials and MOFs will be discussed below. Furthermore, the role of each functional material is also demonstrated. Ni-MOFs possess the function of a glucose oxidation electrocatalyst, and rGO significantly improves the electrochemical properties of Ni-MOFs. In addition, α-CD effectively prevented the aggregation of rGO nanosheets and enhanced the stability of the composites.

#### 3.1.3. MOF/Carbon Nanotubes

Carbon nanotubes (CNTs) are attractive for designing electrochemical sensor components due to their excellent chemical and mechanical properties, good electrical conductivity, and large specific surface area [105,106]. Single-walled nanotubes (SWCNTs) and multiwalled nanotubes (MWCNTs) are the two types of carbon nanotubes according to the arrangement of their graphene cylinders. SWCNTs have only one layer of graphene cylinders and MWCNTs have many layers (about fifty) [107]. Compounding CNTs with other electrochemically active materials is a common approach in glucose sensing technology [108,109]. For example, CNTs act as a support and conductive additive to maintain the mechanical stability and enhance the electrical conductivity of MOFs after continuous measurements, thus ensuring the stability and selectivity of glucose detection [105]. Wu et al. [110] prepared multilayer films of a Cu-MOF/MWCNT non-enzyme glucose sensor by electrodeposition of Cu-MOF crystalline solution and MWNT solution on GCEs multiple times (Figure 3e). Specifically, four layers of Cu-MOF/MWCNT multilayer films on a modified GCE have best electrocatalytic glucose oxidation performance, with a wide linear detection range of 0.5 μM to 11.84 mM, a low detection limit of 0.4 μM and a superior sensitivity of 3878 μA cm^−2^ mM^−1^. Wang and colleagues [111] prepared a layered 3D flower-like Ni-MOF by a solvothermal method, then the synthesized Ni-MOF was combined with SWCNTs by an ultrasonic method. To explore the potential applications of nanocomposites, a Ni-MOF/SWCNT-modified GCE was applied to a non-enzyme electrochemical glucose sensor, which demonstrated that the Ni-MOF/SWCNT-modified GCEs have higher electrochemical response and stronger electrocatalytic activity compared with pristine Ni-MOFs and SWCNTs. P. Arul and colleagues [112] developed a method for preparing 3D nucleated particles (e.g., MPsLCu-MOF) by electrodeposition on a GCE and used it to produce a glucose sensor for human saliva samples (Figure 3f). In the fabrication processes of SWCNTs-MPsLCu-MOF/GCEs, SWCNTs and Cu-MOFs were successively electrodeposited on GCEs. Compared with pristine SWCNTs and MPsLCu-MOF-modified electrodes, the SWCNTs-MPsLCu-MOF/GCE had a higher oxidation current for glucose.

#### 3.1.4. MOF/Carbon Nanohorns

Carbon nanohorns (CNHs) are closed cages made up of C atoms with sp^2^ bonds [113]. Due to their closed cage structure, CNHs can be considered a subset of the high aspect ratio of fullerenes, although CNHs can be opened by oxidation to increase surface area and provide access to the inner cavity. However, their elongated shape means that a closer structural analogue is short SWCNTs, with which they share much common chemistry [114]. In fact, in contrast to CNTs, CNHs have potentially toxic metal catalysts during production and are mass-produced at room temperature. Therefore, CNHs are investigated as an alternative material for nanotubes in a range of fields [115]. In addition, CNHs have the advantages of large surface area, abundant active sites and excellent electrical conductivity [116]. Compared with other carbon-based materials, CNHs also have the advantages of high purity and good dispersion. The above advantages of CNHs lead to believe that CNHs can enhance the performance of MOFs and construct composites with high conductivity, large specific surface area and excellent electrochemical catalytic activity. As shown in Figure 3g, a Cu-MOF/CNH composite was constructed by Zheng and coworkers [117]. The MOF plays a role as an efficient electrochemical catalyst for the electrochemical glucose oxidation, and CNHs can significantly accelerate the electron transfer on the electrode surface. The Cu-MOF/CNHs/GCE has good electrochemical performance for glucose in the range of 250 μM to 1.2 mM, and the detection limit is 78 μM (S/N = 3). As can be seen from Figure 3h, CNHs are loaded on the Cu-MOF surface as a carbon-based functional material instead of serving as the substrate for the growth of MOFs. It can also be seen that CNHs have a rough surface structure, which weakens van der Waals forces, and this is the reason why CNHs are more dispersed in solution than other carbon-based materials.

#### 3.1.5. MOF/Carbon Fibers

Carbon fibers (CFs), as a novel type of fiber material, possess high strength and high modulus of carbon content of over 95%. Currently, polyacrylonitrile (PAN) is the main precursor material for production of CFs, although other precursors (e.g., asphalt and rayon) are also utilized [118]. In addition, CFs not only have the intrinsic properties of carbon materials, but also have the soft machinability of textile fibers. Specially, the combination of MOFs and CFs can not only solve the problems of low conductivity of MOFs but also improve the surface/interface properties of the CFs [119]. Zhao and colleagues [120] developed an effective strategy to induce the growth of hydroxy double salt (HDS) nanosheets on CFs using atomic layer deposition pretreatment, and the HDS nanosheets were chemically converted into ZIF-67 layers and tightly attached to carbon fibers to form composite Co-containing porous smart fibers. Figure 3i shows the schematic diagram of a functional smart fiber for high-performance glucose sensing and a corresponding smart textile, where a dense and uniform functional film is attached to the surface of CFs, forming layered nanostructures with enhanced surface area. The enhanced exposure of the active sites and the improvement of electron transfer in the composite material improve the efficiency of the electrochemical reaction, so that the composite material delivers superior anti-interference ability and fast response ability. Liu et al. [121] prepared a CuCo-MOF with a 2D book-like structure and fixed it onto CF paper for direct electrochemical glucose detection (Figure 3j). The as-prepared 2D book-like CuCo-MOF/CF paper composite shows not only the synergistic effect of bimetallic materials but also unique 2D structural characteristics, which enhance the charge transfer rate at the interface and the electrocatalytic performance. Dey and coworkers [122] supported Ni-MOFs on a flexible carbon nanofiber (CNF) by a simple in situ self-assembly method and used it as a non-binding electrode for non-enzymatic detection of glucose. The porous structure of Ni-MOFs facilitates the close contact between the active center and glucose molecules, thus enhancing the electrocatalytic performance of glucose oxidation, and the high conductivity and large surface area of porous CNFs facilitate electron and mass transfer of the analyte to the Ni-MOF/CNF hybrid sensor.

#### 3.1.6. MOF/Carbon Cloth

Carbon cloth (CC) is an important material made of braided CFs with a diameter of 5 μm to 10 μm [123,124]. It has high porosity (~70%), thus implying a large specific surface area [125]. In addition, it also has the advantages of high conductivity, flexibility, mechanical strength, hydrophobicity and low cost [126,127]. CC’s inherent physical, mechanical and electrochemical properties have recently promoted their use as electrochemical sensors [128]. Thanks to the above advantages, the MOF/CC composites prepared with CC as the support have excellent electrochemical performance [129,130]. Wei et al. [131] prepared a Co-MOF phase with well-aligned 3D nanosheet array architecture on CC (Figure 3i), which can act as an independent Janus catalytic electrode for glucose and water oxidation. The direct growth of Co-MOF nanoarrays on CC not only preserves its inherent molecular metal active sites and micropores, but also gives the Co-MOF advantages such as large specific surface area, high conductivity and efficient electrolyte/catalyst contact. Wei and colleagues [132] reported a Co-MOF/CC/paper hybrid button sensor (Figure 3m) as a portable, robust and user-friendly electrochemical analytical chip for the non-enzymatic quantitative detection of glucose. The flexible Co-MOF/CC sensing interface not only provides sufficient catalytic sites and high specific anisotropy area but also effectively integrates with pattern paper to form electrochemical sensing coin sheets. Xu and coworkers [133] developed an electrochemical non-enzyme glucose sensor though direct growth of bimetallic Ni/Co-MOFs on CC by a simple hydrothermal method. Utilizing synergistic catalysis of Ni and Co elements and excellent electrical conductivity of CC, Ni/Co-MOF/CC electrodes show excellent catalytic activity against glucose with a low detection limit of 100 μM (S/N = 3), fast response time of 2 s and high sensitivity of 3250 μA mM^−1^ cm^−2^.

### 3.2. MOF/Metal-Based Composites

An important way to enhance the conductivity of pristine MOFs and thus improve the sensitivity of them in the electrocatalytic process is to combine the pristine MOFs with metal-based materials, which have good conductivity [134]. In general, metal-based functional materials can wrap outside or be encapsulated inside the pristine MOFs (e.g., metal nanoparticles) or used as substrates to support the pristine MOFs (e.g., metal foam) [41]. Metal nanoparticles (NPs) have received special attention due to their unique electrocatalytic properties and surface plasmon resonance [135]. In particular, surface plasmon resonance peaks are sensitive to the shape and size of metal NPs as well as the dielectric environment. As a result, these NPs have been used in many fields, such as surface-enhanced Raman spectroscopy and electrochemical sensors [136]. Compared to metal NPs, metal foam as a support material provides large surface area, excellent stability and high electrical conductivity [137]. In addition, it overcomes the disadvantage of polymer adhesives in fixing catalysts on conventional electrodes. Combining these metal-based functional materials with the pristine MOFs can enhance the electrical conductivity of pristine MOFs and improve the selectivity of electrochemical sensors.

#### 3.2.1. MOF/Au NPs

**Figure 4:**
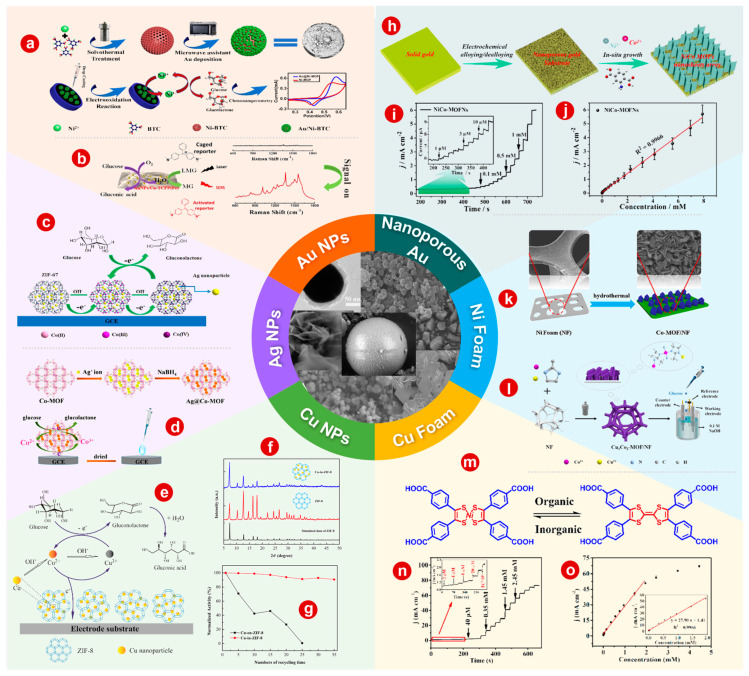
(**a**) Illustrative diagram for preparation of Au NPs@Ni-MOF as well as the proposed mechanism for non-enzymatic oxidation of glucose at Au NPs@Ni-MOF surface in alkaline media. (**b**) Schematic diagram of the non-enzymatic tandem reaction strategy for SERS detection of glucose. (**c**) Schematic diagram of glucose electrooxidation to gluconolactone in NaOH aqueous solution over GCE modified with Ag NPs@ZIF-67. (**d**) Illustrative diagram for synthesis of modified GCE and its application in electrocatalyst of glucose. (**e**) Schematic diagram for glucose oxidation over SPCE modified with Cu-in-ZIF-8. (**f**) XRD spectra of the ZIF-8 from simulation and preparation, as well as the Cu-in-ZIF-8. (**g**) Catalytic activity changes in the Cu-in-ZIF-8 catalyst and the Cu-on-ZIF-8 catalyst with recycling time. (**h**) Schematic diagram of the synthetic process for vertical growth of NiCo-MOF nanosheet matrix (nanoporous Au from etching of Au metal was used as the substrate). (**i**) Amperometric response curve of NiCo-MOFN electrode upon stepwise glucose addition into 0.1 M of NaOH solution under voltage of 0.5 V versus SCE; the inset shows a magnified view of the area marked with green. (**j**) Calibration curve of the amperometric response for the fabricated electrode. (**k**) Schematic diagram of the Co-MOFN matrix formation on Ni foam. (**l**) Illustrative diagram for fabricating CuCo-MOF/Ni foam and detecting glucose on the as-fabricated electrode. (**m**) Structural description of [Ni(C_2_S_2_(C_6_H_4_COOH)_2_)_2_] as well as the H_4_TTFTB. (**n**) Amperometric response curve of 1-Cu foam upon stepwise glucose addition into 0.1 M of NaOH aqueous solution (inset: Electrode current response to the glucose addition of 2-40 μM). (**o**) Relevant calibration curve of 1-Cu foam electrode upon stepwise glucose addition into 0.1 M of NaOH under voltage of 0.65 V. The figures are Reprinted with corresponding permission as follows: (**a**): [57] Copyright 2020, Springer. (**b**): [142] Copyright 2020, American Chemical Society. (**c**): [143] Copyright 2018, Elsevier. (**d**): [144] Copyright 2019, American Chemical Society. (**e**–**g**): [146] Copyright 2016, Elsevier. (**k**): [150] Copyright 2019, Elsevier. (**l**): [151] Copyright 2022, Elsevier. (**m**–**o**): [153] Copyright 2020, American Chemical Society.

Au NPs have stable chemical properties and strong surface plasmon resonance characteristics and have always been the focus of research on metal NPs [138,139]. In particular, spherical Au NPs have precise surface plasmon resonance spectra without undesirable surface plasmon resonance signals [140]. Hang et al. [141] successfully synthesized Au@MIL-100 NP arrays by a facile solvothermal method. The Au@MIL-100 NP arrays were characterized by two peaks in the visible spectrum. The first peak represents the surface plasmon resonance of the Au nanospheres, while the other, the diffraction peak, arises from the periodic structure in the NP arrays. The diffraction peak responds sensitively to low glucose concentration, while the surface plasmon resonance peak responds rapidly to a high concentration. Chen and colleagues [57] decorated Au NPs on a Ni-MOF to enhance its electrochemical performance for non-enzymatic glucose detection (Figure 4a). The prepared Au@Ni-MOF sensor has high glucose detection performance, with a wide linear range of 5 to 7400 μM, high sensitivity of 1447.1 μA mM^−1^cm^−2^ and low detection limit of 1.5 μM. Furthermore, Hu and coworkers [142] prepared a multifunctional S-hybrid nanosheet by an in situ modification of Au NPs onto a 2D Cu-MOF (Figure 4b). In the presence of Au NPs, sensitive and selective surface-enhanced Raman scattering for the determination of glucose is realized. On the basis of Au NPs/Cu-MOFs, an enzyme-free surface-enhanced Raman scattering detection assay was used to detect glucose in saliva, and the recovery rate increased from 96.9 to 100.8%.

#### 3.2.2. MOF/Ag NPs

As Ag NPs have the advantages of high conductivity and biocompatibility, an interest in the design of electrochemical sensors containing Ag has been stimulated. Meng et al. [143] prepared an Ag NPs@ZIF-67 nanocomposite by a sequential deposition reduction method. The Ag NPs@ZIF-67/GCE delivers strong catalytic activity for glucose oxidation. The electrocatalytic performance of Ag@ZIF-67 has been studied, and the results show that the response time of the modified electrode is more than two times shorter and the sensitivity is increased by two and a half times as silver content increases from 0% to 0.5%. In addition, the mechanism of glucose oxidation by Ag NPs@ZIF-67 nanocomposite has also been studied, as shown in Figure 4c. Liu and colleagues [144] designed and prepared a 3D porous Co-MOF with pentanuclear Co(II) clusters by using oxygen bridging 5,5 ′-oxyphthalic acid (H4L) ligands. After that, Ag NPs were encapsulated by a deposition–reduction method to obtain Ag NPs@Co-MOF composite (Figure 4d). The Ag NPs@Co-MOF composite-modified GCE has obvious electrocatalytic activity for glucose oxidation, with remarkable sensitivity and selectivity, good stability and low detection limit.

#### 3.2.3. MOF/Cu NPs

Cu NPs are particularly attractive due to the high natural abundance and low cost of copper, as well as the practical and straightforward variety of methods for preparing Cu-based nanomaterials [145]. In addition, because Cu has a wide range of oxidation states (Cu^0^, Cu^I^, Cu^II^ and Cu^III^) that allow it to react through both single-electron and two-electron pathways, Cu-based materials can facilitate and experience a variety of reactions [66]. Due to the advantages of Cu-based materials, the combination of Cu NPs and MOFs is one of the important ways to enhance the electrochemical performance of the pristine MOFs. Shi and colleagues [146] encapsulated Cu NPs in ZIF-8, which was used as a porous matrix for encapsulation, for non-enzymatic glucose sensing in alkaline media and demonstrated the electrocatalytic oxidation mechanism of glucose on the surface of SPCEs modified by Cu-in-ZIF-8 (Figure 4e). XRD patterns show that the data of the synthesized ZIF-8 are in good agreement with the simulated data of ZIF-8 (Figure 4f). For Cu-in-ZIF-8, the diffraction peaks of ZIF-8 can be well identified, indicating that the crystal structure of ZIF-8 remains basically unchanged after the successful encapsulation of Cu NPs. In addition, ZIF-8, as a substrate for encapsulating Cu NPs, can also protect Cu NPs from dissolution and aggregation during electrocatalytic processes. As shown in Figure 4g, the electrochemical properties of Cu-on-ZIF-8 have also been studied. It is found that Cu-in-ZIF-8 has higher activity and better stability for glucose cycling tests in alkaline media.

#### 3.2.4. MOF/Nanoporous Au

**Figure 5:**
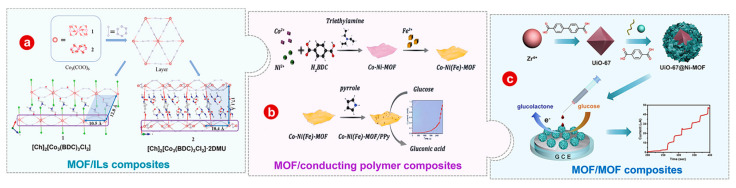
**(a**) The structural constructions of [Ch]_2_[Co_3_(BDC)_3_Cl_2_] as well as its derivative [Ch]_2_[Co_3_(BDC)_3_Cl_2_]·2DMU. (**b**) Illustrative diagram of synthesis of Co-Ni(Fe)-MOF/PPy. (**c**) Illustrative diagram of preparation of core–shell structure UiO-67@Ni-MOF composite with PVP-regulated internal growth and glucose sensing with electrode modified by UiO-67@Ni-MOF. The figures are Reprinted with corresponding permission as follows: (**a**): [157] Copyright 2021, Royal Society of Chemistry. (**b**): [160] Copyright 2022, Elsevier. (**c**): [163] Copyright 2020, Elsevier.

Nanoporous Au needles have previously been shown to be an economical, efficient, electrically conductive substrate for sensitive glucose detection [147]. Li and colleagues [148] used nanoporous Au as an effective substrate to guide the 2D vertical growth of NiCo-MOFs (NiCo-MOFNs) without causing nanostructure accumulation (Figure 4h). Figure 5a shows the amperometric response curves of the NiCo-MOFN electrodes continuously added into 0.1 M of NaOH aqueous solution under a voltage value of 0.5 V. In addition, the relevant calibration curves are shown in Figure 4j, where a linear relationship was observed. These results suggest that the NiCo-MOFN nanocomposite electrode has a good linearity ranging from 0.0010 to 8 mM as well as a low limit of detection of 0.29 μM (S/N = 3).

#### 3.2.5. MOF/Ni Foam

Ni foams (NFs) could also be applied as support materials for MOFs, providing good stability, large surface area and superior electrical conductivity [149]. Li and coworkers [150] proposed a one-pot hydrothermal synthesis approach for a Co-MOF nanosheet matrix with NF as the support (Figure 4k). The synthesized Co-MOF/NF composite was highly active in the electrochemical oxidation of glucose in alkaline media with a sensitivity of 10,886 μA mM^−1^ cm^−2^ and a detection limit of 1.3 μM (S/N = 3). Furthermore, Du and colleagues [151] grew CuCo-MOFs on the surface of NF by a hydrothermal method (Figure 4l) and achieved excellent sensitivity through the combination of the advantages of Cu/Co cations and MOFs. At a molar ratio of 1:2 for Cu^2+^ and Co^2+^, a high sensitivity level as well as low value of detection limit for the CuCo-MOF/NF electrode were observed, which were 8304.4 μA mM^−1^ cm^−2^ and 0.023 μM (S/N = 3), respectively.

#### 3.2.6. MOF/Cu Foam

Cu foam also has broad application prospects in qualitative and quantitative chemical analysis [152]. Zhou et al. [153] synthesized [Mn_2_{Ni(C_2_S_2_(C_6_H_4_COO)_2_)_2_}(H_2_O) _2_]·2DMF (1, herein DMF represents N,N-dimethylformamide) on Cu foam by a one-step hydrothermal method. As a H_4_TTFTB analog, inorganic [Ni(C_2_S_2_(C_6_H_4_COO)_2_)_2_] composite was synthesized successfully for use as novel building blocks (Figure 4m). It is an important linker with high redox activity and multipurpose function in the preparation of new functional MOFs. Figure 4n is a representative current–time graph of the 1-Cu foam electrode with a continuous stepwise change in the concentration of glucose. The inset of Figure 4n shows the low glucose concentration ranging from 2.0 × 10^−6^ to 4.0 × 10^−5^ M. As shown in Figure 4o, the curve has a linearity ranging from 2.0 × 10^−6^ to 2.0 × 10^−3^ M, as well as a sensitivity and a limit of detection of 27.9 A/M·cm^2^ and 1.0 × 10^−7^ M (S/N = 3), respectively.

### 3.3. MOF/Other Types of Functional Materials

#### 3.3.1. MOF/Ionic Liquids

Modifying the MOF structure by immobilizing ionic liquids (ILs) may lead to better performance and provide a more reactive catalytic system [154]. Recently, it has been proved that introducing ILs can improve the adsorption performance of MOFs to chemical substances [155], which is conducive to promoting catalysis. In addition, the introduction of ILs into the synthesis of MOFs can improve their electrical conductivity, thus enhancing their electrocatalytic performance. Zhang et al. [156] prepared a Co-MOF/IL composite though an ionothermal strategy. The Co-MOF has a framework structure of [BMI]_2_[Co_3_(BDC)_3_Br_2_], and the [BMI]^+^ is anchored in the interlayer space. The electrode modified with Co-MOF/IL composite has a broad linearity ranging from 5 to 900 μM as well as a detection limit as low as 1.6 μM (S/N = 3). Using deep eutectic solvent of choline chloride (ChCl) and 1,3-dimethylurea (DMU), Ma and coworkers [157] prepared [Ch]_2_[Co_3_(BDC)_3_Cl_2_] as well as its derivative [Ch]_2_[Co_3_(BDC)_3_Cl_2_]·2DMU by an ionothermal strategy. The structures of the two Co-MOF/IL composites are shown in Figure 5a. Many [Ch]^+^ are embedded in the 12.8 Å interlayer space and act as structural templates and charge balancers. Among the two Co-MOF/IL composites, [Ch]_2_[Co_3_(BDC)_3_Cl_2_]/GCE has a better current response, so it is used as an electrocatalyst. The linear range of [Ch]_2_[Co_3_(BDC)_3_Cl_2_]/GCE is 10 to 1200 μM, and the limit of detection is 3.2 μM (S/N = 3) in 0.01 M NaOH.

#### 3.3.2. MOF/Conducting Polymer

Conductive polymers have been used in the field of electrochemical sensing because of their excellent electrical performance [158]. Among them, conducting polypyrrole (PPy) has been widely used in a variety of non-enzymatic glucose sensing nanocomposites [159]. Chen and colleagues [160] prepared a Co-Ni(Fe)-MOF 2D nanosheet composite and assembled it with conductive PPy by a simple strategy for electrochemical non-enzymatic glucose detection (Figure 5b). Due to the introduction of conductive PPy, the prepared modified electrode shows an enlarged number of active sites as well as superior performance of the electron transfer, achieving a sensitivity value as high as 1805 μA/mM cm^2^ and a limit of detection of 1.13 μM (S/N = 3).

#### 3.3.3. MOF/MOF

Integration of MOFs of different types is also a promising method for functionalization of the individual MOFs [161,162]. In this case, Lu et al. [163] developed an electrochemical non-enzymatic sensor for glucose detection in alkaline media based on the core–shell structured composite of MOF@MOF (Figure 5c). Additionally, a UiO-67@Ni-MOF composite was also prepared using the stretching growth of UiO-67 under the regulation of polyvinylpyrrolidone. The speed of the electron transfer in this composite is promoted by the superior conductivity and the large surface area characteristics of the UiO-67 material. On the other hand, Ni-MOF can be used as an electrocatalyst because of its good activity for electrochemically oxidizing glucose. The UiO-67@Ni-MOF/GCE was observed to have a short response time below 5 s, broad linearity ranging from 5 to 3.9 mM and a low detection limit of 0.98 μM.

### 3.4. MOF/Two or More Kinds of Functional Material

The electrochemical property of MOFs compounded with one kind of functional material still fails to meet the actual needs. More and more studies focus on composites of MOFs with two or more kinds of functional material. Wang et al. [164] developed a biomimetic atomized MOF microelectrode with a non-enzymatic sweat biosensor induced by the electrochemical anode interface. The preparation process of the biomimetic atomized MOF microelectrode comprises construction of a 3D graphene framework wrapped with activated carbon fibers (ACFs) as a pattern axis and then electrodeposition of Cu nanosponges onto the graphene skeleton surface, where an in situ self-assembly process of a mist structure of Cu(INA)_2_ is subsequently induced. This nanoscaled MOF films with the coadjacent channels provide increased exchange/transfer rates of electrons and mass in the electrochemical reactions due to the span of stratified length scales. Arif and coworkers [165] developed a sensitive and effective glucose sensor with a ZIF-67 composed of Ag@TiO_2_ nanoparticles for detecting glucose under alkaline conditions. Excellent performances were detected for this Ag@TiO_2_@ZIF-67/GCE sensor, and its sensitivity, linear concentration range, response time and detection limit values are 0.788 µA µM^−1^ cm^−2^, 48 µM^−1^ mM, 5 s and 0.99 µM (S/N = 3), respectively. Chen et al. [70] selected Co- and Fe-ZIF materials to prepare classical 2D MOF structures. ZIF/NF composites were synthesized by in situ growth of foliated ZIFs onto the NF surface. They were then easily converted in situ as a precursor to PBA/ZIF/NF complexes by using a hydrothermal approach, where ZIF has a dual function of supporting the scaffold and the metal source as a sacrificial template. CoFe-PBA/Co-ZIF/NF shows excellent properties for sensing glucose with the linearity ranging from 1.4 μM to 1.5 mM. Its sensitivity and detection limit are 5270 μA/mM cm^2^ and 0.02 μM (S/N = 3), respectively. Elizbit and colleagues [166] used porous ZIF-67 material for encapsulation of Ag nanoparticles and modified the ZIF-67 surface with MWCNTs for preparation of Ag@ZIF-67/MWCNT nanocomposites. Subsequently, the obtained Ag@ZIF-67/MWCNT nanocomposites were loaded onto NF for electrochemical sensing of glucose. In a glucose concentration range of 33–400 μM, Ag@ZIF-67/MWCNT/NF had high electrocatalytic activity and a low limit of detection of 0.49 μM (S/N = 3). Dong et al. [167] developed a flexible composite microelectrode coated with CFs based on a Ni-MOF nanosheet array supported by rGO and investigated its practical performance for glucose detection. Due to the synergistic effect between the conductive carbon substrates and the high loading density of Ni-MOF nanoflakes, this microelectrode of Ni-MOF/rGO/CF has a superior activity for electrochemically detecting glucose. It shows a high level of sensitivity of 852 µA/cm^2^ mM as well as a linearity ranging widely from 6 µM up to 2.09 mM.

## 4. Conclusions and Perspective

The non-enzyme electrochemical glucose sensor shows characteristics of rapid analysis, high sensitivity and specificity. It is a suitable technology for glucose sensing. In addition, non-enzymatic electrochemical glucose sensors are a suitable choice for direct electrocatalysis based on electrode materials. The large surface area and the superior chemical tunability characteristic of MOFs enhance their electrocatalytic activity, offering potential application in non-enzymatic electrochemical detection of glucose. The poor electrical conductivity and low stability of pristine MOFs are major concerns in the related research. Generally, the low stability against water for MOFs can be attributed to the ligand bonds of the metal ligands being easily cleaved by water molecules. Compounding the pristine MOFs with other functional materials is one of the most important ways to solve their inherent defects. In this review, applications of pristine MOFs (monometallic MOFs and bimetallic MOFs) and MOF composites in electrochemical non-enzymatic detection of glucose were summarized. For the purposes of electrochemical sensing properties of the pristine MOFs (monometallic and bimetallic) and MOF composites, the materials described are listed in Table 1. Despite the wide range of applications of MOFs in the area of interest, a number of practical challenges remain. Here are our views on some of the key challenges and solutions for future developments in this area:

(1) A variety of simple and effective synthesis methods (such as one-pot methods) have been gradually developed and perfected to make large-scale synthesis of the MOF and its composites possible. However, there is still a need to replace the precious metals used in existing MOF@metal NPs with low-cost but highly reactive metal NPs.
(2) Aiming at improving the water stability of MOF s and MOF composites and reducing the content of other solvent molecules in the coordination center, it is necessary to develop a green way to synthesize MOFs and MOF composites in aqueous solution.
(3) To gain further insight into the electrochemical mechanisms, in-depth studies through in situ test and theoretical simulations are necessary. In addition, advanced characterization instruments are necessary.
(4) MOFs can be combined with nanofiber paper, portable fluorescence detectors and smartphones for development of convenient, quick and accurate electrochemical sensing techniques/equipment. Fluorescent detectors and smartphones are combined to develop simple, fast, accurate and portable methods of electrochemical sensing.

**Table 1:** The sensing properties of materials mentioned in the references in this review.

Affiliation	Materials	Linear Range (µM)	Sensitivity (µA mM^−1^ cm^−2^)	Detection Limit (µM, S/N = 3)	Reference
Monometallic MOFs	ZIF-67 HNPs	50 to 3300	445.7	0.96	[56]
	Ni-based MOF	10 to 800	635.9	6.68	[61]
	Ni-MIL-77 NBs	1 to 500	1.542	0.25	[62]
	CPO-27-Ni^II^	40 to 6000	40.95 (µA mM^−1^)	1.46	[63]
	Ni-based MOF	10 to 400	3297.1	8.97	[64]
	Cu-based MOF	2 to 1400 1400 to 4000	1044 682	0.6	[68]
	Cu-based MOF	5 to 3910	/	0.11	[69]
Bimetallic MOFs	CoZn-BTC	1 to 255 255 to 2530	1218 510	4.7	[78]
	ZIF67/ZIF8	50 to 5000	833.61	6.5	[79]
	E-NiCo-BTC	1 to 1780 1780 to 5030	1789 1436	0.187	[80]
	NiCoBP-Br	0.5 to 6065.5	1755.51	0.0665	[81]
	Ni@Cu-MOF	5 to 2500	1703.33	1.67	[82]
	NiCu-MOF-6	20 to 4930	1832	15	[83]
	Ni3Zn-MOF	0.5 to 5065	512.53	0.125	[84]
MOF/ carbon-based composites	Co-MOF/EG	1 to 3330	23 (µA mM^−1^)	0.58	[99]
	NH_2_-GP-Cu-MOF	0.05 to 1775.5	5.36	0.03	[33]
	Co_3_(BTC)_2_/rGO	1 to 330 330 to 1380	1702 1002	0.33	[103]
	α-CD-rGO/Ni-MOF	0.65 to 4828 4828 to 9178	1385 760	0.3	[104]
	Ni-MOF/CNTs	1 to 1600	13.85	0.82	[105]
	Cu-MOF/MWNTs	0.5 to 11,840	3878	0.4	[110]
	Ni(TPA)-SWCNT	20 to 4400	/	4.6	[111]
	SWCNTs-MPsLCu-MOF	0.02 to 80	573	0.00172	[112]
	Cu-MOF/CNHs	0.25 to 1200	/	0.078	[117]
	Co-PSF	0.5 to 30	4835	0.3	[120]
	CuCo-MOF/CFP	1 to 1200	6861	0.12	[121]
	Ni(PDA)MOF@CNF	10 to 3000	9457.5	0.053	[122]
	Co-MOF NS/CC	4 to 4428	1113	1.2	[131]
	Co-MOF/CC	800 to 16,000	/	150	[132]
	Ni/Co(HHTP)MOF/CC	0.3 to 2312	3250	0.1	[133]
MOF/ metal-based composites	Au@Ni-BTC	5 to 7400	1447.1	1.5	[57]
	Au NPs/Cu-TCPP	160 to 8000	/	3.9	[142]
	Ag@ZIF-67	2 to 1000	0.379	0.66	[143]
	Ag@ Co(II)-based 3D porous MOF	5 to 550	0.135	1.32	[144]
	Cu-in-ZIF-8	0 to 700	412	2.76	[146]
	NiCo-MOF/NPG	1 to 8000	684.4	0.29	[148]
	Co-MOF/NF	1 to 3000	10,886	0.0013	[150]
	Cu_1_Co_2_-MOF/NF	50 to 500	8304.4	23	[151]
	1@Cu Fo	2 to 2000	27,900	0.1	[153]
MOF/ ionic liquids	Co-MOF/IL	5 to 900	169	1.6	[156]
	[Ch]_2_[Co_3_(BDC)_3_Cl_2_]	10 to 1200	160.75	3.2	[157]
	[Ch]_2_[Co_3_(BDC)_4_]·2DMU	10 to 1200	71.71	3.2	[157]
MOF /conducting polymer	Co-Ni(Fe)-MOF/PPy	2 to 3000	1805	1.13	[160]
MOF@MOF	UiO-67@Ni-MOF	5 to 3900	/	0.98	[163]
MOF/ two or more kinds of functional material	ACF-rGO/Cu(INA)_2_	25 to 16,875	46.8	0.5	[164]
	Ag@TiO_2_@ZIF-67	48 to 1000	788	0.99	[165]
	CoFe-PBA/Co-ZIF/NF	1.4 to 1500	5270	0.02	[70]
	Ag@ZIF-67/MWCNT	33 to 400	13.014	0.49	[166]
	Ni-MOF/rGO/CF	6 to 2090	852	0.6	[167]

In summary, the further improvement in performances of the non-enzymatic electrochemical glucose sensors of pristine MOFs by introducing functional materials has important implications. MOF composites, although still presenting some challenges, have seen rapid development in recent years and the implementation of novel functional materials is imminent. This study provides not only further insight into MOFs and MOF composites as promising alternative materials for application in non-enzymatic electrochemical glucose sensing but also valuable references for future research.

## Data Availability

Not applicable.
